# Cell based therapy in Parkinsonism

**DOI:** 10.1186/2047-9158-2-13

**Published:** 2013-06-04

**Authors:** Johannes PJM de Munter, Chongsik Lee, Erik Ch Wolters

**Affiliations:** 1 Department of Neurosciences University Maastricht, Maastricht, The Netherlands; 2 Amarna Stem Cells Group, Maastricht, The Netherlands; 3 Department of Neurology, Asan Medical Center University of Ulsan, Seoel, South Korea; 4 Department of Neurology, UniversitatsSpital, Zurich, Switzerland

**Keywords:** Adult stem cells, Parkinson’s disease, Multiple system atrophy, BDNF, GDNF, Expanded MSC, Preclinical

## Abstract

Parkinson’s disease (PD) is a synucleinopathy-induced chronic progressive neurodegenerative disorder, worldwide affecting about 5 million humans. As of yet, actual therapies are symptomatic, and neuroprotective strategies are an unmet need. Due to their capability to transdifferentiate, to immune modulate and to increase neuroplasticity by producing neurotrophic factors, adult stem cells (ASC) might fill this gap. Preclinical research in 6-hydroxydopamine (6-OHDA) and/or 1-methyl-4-phenyl-1,2,3,6-tetrahydropyridine (MPTP) lesioned animals established persistent improvements of motor behavior after ASC-treatment. Histological/histochemical measurements in these animals evidenced an intracerebral applied ASC-induced increase of Tyrosine Hydroxylase-positive (TH^+^) cells with increased striatal dopamine levels, suggesting cell rescue. Likewise, clinical experience with subventricular applied ASCs in PD patients, although limited, is encouraging, evidencing neurorescue especially during the early phase of the disease. In multiple system atrophy (MSA) or progressive supranuclear palsy (PSP) patients, though, only marginal reduced progression of natural progression could be established after subventricular or intravasal ASC implantations.

## Introduction

Parkinson’s disease (PD) is the most common chronic progressive neurodegenerative disorder after Alzheimer’s disease [1], world-wide affecting nearly 5 million people aged 50 years or more, and expected to double over the next 20 years [2]. It comes with a twofold higher mortality rate, mainly due to pneumonia, shortening life expectancy with nearly 10 years [3,4]. The result of the α-synucleinopathic degeneration of the nervous system, starting in the peripheral nervous system and lower brainstem and progressively extending over the upper brainstem and neocortex, symptomatology in PD comprises dysfunctions of the whole nervous system. It may start with a range of non-motor symptoms such as disorders of the autonomic nervous system, olfaction, sleep, mood and subtle cognitive deterioration, before a degeneration of the dopamine producing cells in the upper brainstem (nigral substance) may manifest with motor parkinsonism, the clinical hallmark of this disease, and way before involvement of the neocortex induces dementia [5]. PD is mainly recognized when first symptoms of motor parkinsonism (hypokinesia, bradykinesia, rigidity, tremor and the loss of postural reflexes) develop as the result of the loss of the majority of the dopaminergic neurons of the pars compacta of the substantia nigra with a striatal dopaminergic depletion of over 80% [6]. As of yet, treatment in PD is based on the pulsatile (oral) or continuous (subcutaneous, intrajejunal) suppletion of the striatal dopamine deficiency with dopamine agonists and/or the dopamine precursor levodopa, mostly in combination with a peripheral dopa decarboxylase inhibitor and/or in combination with inhibitors of mono-amine oxidase B (MAO-B) and/or catechol-O-methyl transferase (COMT), in order to restore striatal dopaminergic denervation [7].

Actual therapy only symptomatically affects motor parkinsonism, though. Therapies affecting non-motor symptomatology, and above all protective or restorative treatments are unmet needs in PD. In order to reach these needs, recently, experiments with cell based therapies to rescue or replace dopamine-secreting cells, or with cells able to secrete paracrine factors modulating brain tissue repair were initiated [8-12].

In this review, these experimental stem cell based therapeutic strategies will be discussed. As the application of embryonic stem cells and induced pluripotent stem cells comes with an unacceptable risk of tumor induction [13-16], this review will only cover experiments dealing with expanded, whether or not genetically modified, autologous or allogenic bone marrow-derived and/or neural progenitor stem cells.

### Adult stem cells (ASC)

Adult stem cells comprise mesenchymal stem cells (MSCs), hematopoietic stem cells (HSCs) and ectodermal stem cells (ESCs). The majority of the cited preclinical and clinical studies use expanded and/or induced mesenchymal stem cells.

Re-implanted adult autologous stem cells, easily harvested out of the iliac crest and whether or not expanded, as a rule, will migrate towards diseased tissue, a phenomenon called homing [17,18]. Those stem cells have the potency to modulate immune responses [19,20] and to both transdifferentiate into target cells in order to replace damaged cells [21-24], and secrete paracrine (trophic) factors relevant for cell protection and cell repair by the inhibition of apoptotic pathways [25-27]. So, even before differentiation [28,29], mesenchymal stem cells, might express brain-derived neurotrophic factor (BNDF), glial cell-derived neurotrophic factor (GDNF) and stromal-derived factor (SDF-1α). BDNF is shown to have a neuroprotective effect on cultured rodent neurons via the Pl_3_kinase/Akt pathway by inhibiting neural death initiated by trophic factor withdrawal or by the exposure to nitric oxide [30]. GDNF provides neural protection against proteasome inhibitor-induced dopamine neuron degeneration [31], although its biological effect on the clearance of mature formed α-synuclein aggregation could not be observed, probably due to its short duration of administration [31]. SDF-1α, in low doses, promotes dopamine release from 6-OHDA-exposed PC12 cells (cell line derived from a pheochromocytoma), presumably by preservation and enhanced survival of these cells, as these phenomena are blocked by administration of anti-SDF-1α antibodies [32]. A high concentration of SDF-1α, however, rather enhances apoptosis [33]. SDF-1α acts through CXCR4 (chemokine receptor type 4) resulting in a down regulation of caspase-3 and an activation of the PI3/Akt pathway [34]. SDF-1α also enhances the survival of neural progenitor cells through the receptors CXCR7 and CXCR4 by up regulation of the ERK1/2 (Mitogen-Activated Protein kinase 3) endocytotic signaling pathway [35].

The route of administration (intravasal, intraparenchymal) during the re-implantation of the stem cells seems to have a major impact on the specific transdifferentiation and/or secretion patterns of them, as the actual environment influences the further developments of these cells. However, by inducing stem cells before re-implantation it is also possible to induce these developments already in vitro. Indeed, by exposing these cells to trophic factors, including epidermic growth factor (EGF) and basic fibroblast growth factor (bFGF) [36], by transducing them with a viral vector, and/or by binding them to pharmacologically active microcarriers, containing trophic factors such as NT-3 (Neurotrophin-3) [37], it is possible to further differentiate the stem cells, prior to the re-administration to the target organs [38,39]. So, in vitro, adult stem cells may be predisposed to differentiate into dopamine producing cells [21,40], thus offering a potential alternative for dopamine substitution therapies, or into cells secreting neuroprotective and/or neurorestorative trophic factors, thus protecting for dopaminergic cell death respectively stimulating neurorestoration [41].

Due to the lung trap [42] and the blood–brain-barrier [43,44], intravasal application of stem cells for disorders of the central nervous system is suggested less effective as compared to intracerebral administration [23,45].

### Preclinical experience with ASC in motor parkinsonism

In experimental animals, motor parkinsonism (but not PD) might be induced by intra-nigral 6-hydroxydopamine (6-OHDA) as well as by subcutaneous carbobenzoxy-leu-leu-leucinal (MG-132) and/or subcutaneous or intravasal 1-methyl-4-phenyl-1,2,3,6-tetrahydropyridine (MPTP) [46,47]. MPTP models have the advantage that the lesions are induced through a less invasive (subcutaneous or intravasal) route of administration as compared with the stereotactic surgical approach when applying 6-OHDA. To optimally investigate neural protection, the non-human primate MPTP model seems to be preferred [46]. Tables 1 and 2 present the results of preclinical interventions using cell-based strategies in the 6-OHDA, MPTP and the proteasome inhibitor MG-132 animal models. In all the presented animal studies immune suppression was used to prevent rejection of the cells. However, the use of immune suppression may influence not only the behavior of the Stem Cells [48] but also the severity of the lesions in animal models [49,50].

**Table 1:** Placebo-controlled stem cell applications in rodent animal models of parkinsonism

***Striatal 6-OHDA lesioned rats***				
**Authors animals (Sprague–Dawley rats)**	**Product expanded allogenic ASCs Cyclosporin A**	**Applic/Observ time**	**Characteristics/Dosages/Application**	Outcome (* = p < 0.05/** = p < 0.01/*** = p < 0.001)
Bouchez, Sensebe et al. [51]	rMSCs	14/35 days	1. No intervention (n = 6)	• Rotational behavior (turns/min)
			2. Intrastriatal saline (n = 7)	1. No-intervention group: 23.8 ± 2.1
			3. Intrastriatal 1.8×10^5^ rMSCs (n = 7)	2. Control saline-treated group: 25.1 ± 1.7
Female rats	riMSCs		4. Intrastriatal 1.8×10^5^ riMSCs (n = 7)	3. MSC-treated group: 14.1 ± 3.3*
				4. Enriched rMSC-treated group: 10.8 ± 1.7*
				• TH-positive neurons:
				1. No-intervention group: 24.2 ± 6.7%
				4. Enriched riMSC-treated group: 52.5 ± 8.2%*
Wang, Yasuhara et al. [32]	Fibroblasts	2 hr/28 days	1. Intravenous saline (n = 7)	• Amphetamine-induced rotational behavior
			2. Intravenous 10^7^ fibroblasts (n = 6)	1. Control group: 8.5 ± 3.5 turns/min
Female rats	rMSCs		3. Intravenous 10^7^ rMSCs (n = 6)	2. Fibroblast group: 8.2 ± 3.3 turns/min
				3. rMSC group: 1.2 ± 0.7 turns/min*
				• Cylinder test
				1. Control group: 64.7 ± 17.3%
				2. Fibroblast group: 60.2 ± 16.1%
				3. rMSC group: 29.3 ± 13.7%*
				• Preservation of TH^+^cells: 3* > 2 > 1
Danielyan, Schafer et al. [53]	rMSCs (EGFP labeled)	7,9/110–136 days	Intranasal saline day 7 and 9 (n = 7)	• Stepping ratio (contralateral/ipsilateral) MSC-treated group 2 (0.7)** > group 1 (0.1)
			1. Intranasal 5x10^5^ MSC day 7 and 9 (n = 9)	• Amphetamine-induced rotational behavior
				MSC-treated group 4* < group 3
Female rats			2. Intranasal saline day 7 and 9 (n = 10)	• Histology
			Intranasal 5×10^5^ MSC day 7 and 9 (n = 12).	a. Group 4: 24% of MSCs survived in central nervous system for at least 4.5 months
				b. TH^+^ cell survival: Group 2* > 1 and 4* > 3
				c. Inflammatory cytokines Group 2* < 1 and 4* < 3
Blandini, Cova et al. [58]	hMSCs	5/28 days	1. Intrastriatal saline (n = 9)	• Apomorphine-induced rotational behavior
Male rats			2. Intrastriatal 1×10^5^ hMSCs (n = 8)	1. No effect
				2. Reduced rotational behavior*
				• Expression of GDNF increased in hMSCs group
				• Apoptosis decreased in hMSCs treated group
Cova, Armentero et al. [57]	hMSCs	5/28 days	SHAM unilateral lesion	• Dose-dependent neurorescue effects (hMSCs vs saline) in unilateral 6-OHDA lesioned, but not SHAM lesioned, rats with
			1.Intrastriatal saline	
			2. Intrastriatal 3.2×10^4^ hMSCs (n = 6-10)	
Male rats			3. Intrastriatal 1.8×10^5^ hMSCs (n = 6-10)	a) Reduction*^/^** ongoing toxin-induced degeneration of dopaminergic terminals
			6-OHDA unilateral lesion	
			1. Intrastriatal saline	b) Enhanced neurogenesis*^/^** (neural progenitor cells) in the periventricular zone
			2. Intrastriatal 3.2×10^4^ hMSCs (n = 6-10)	
			3. Intrastriatal 1.8×10^5^ hMSCs (n = 6-10)	c) Persistent release of specific cytokines
Delcroix, Garbayo et al. [37]	rMSCs	14/64 days	1. Intrastriatal saline (n = 6)	• Rotational behavior (turns/min):
			2. Intrastriatal 1.5×10^5^ rMSCs (n = 6)	1. saline treated group: 18.5
			3. Intrastriatal 1.5×10^5^ riMSCs + P (n = 6)	2. rMSCs-treated group: 17.5
Female rats	riMSCs		4. Intrastriatal 1.5×10^5^ riMSCs + P + NT3 (n = 6)	3. riMSCs + P treated group: 8.5*
				4. riMSCs + P + NT3 treated group: 3.0*
	riMSCs + P			• Preservation of TH^+^cells : 4* > 3 > 2 > 1
Levy, Bahat-Stroomza et al. [56] Male rats	hMSCs	35/125 days	1) Intrastriatal saline in 5 (n = 7)	• Rotational behavior (turns/min) (post-lesional 100%)
	hiMSCs (neural phenotype)		2) Intrastriat. 5×10^5^ MSC’s (n = 7)	1) saline-treated group: 88%
			3) Intrastriat. 5×10^5^ neural iMSC’s (n = 7)	2) hMSCs-treated group: 90%
				3) hiMSCs-treated group: 42%*
Sadan, Bahat-Stromza et al. [55]	hMSCs	1 hr/42 days	1) Intrastriatal saline (n = 10)	• D-amphetamine-induced rotational behavior
Male rats	hiMSCs (BDNF/GDNF)		2) Intrastriatal 1.5 or 4.5×10^5^ hMSCs (n = 21)	1. saline group: increase 4.74 ± 1.07 turns/min
				2. MSCs group: increase 2.86 ± 0.54 turns/min
			3. Intrastriatal 1.5 or 4.5×10^5^ hiMSCs (n = 21)	3. iMSCs group: increase 2.16 ± 0.37* turns/min
				• TH-positive area (treated versus untreated site)
				2. hMSCs group: treated site > untreated site
				3. hiMSCs group: treated site* > untreated site
Zhu, Ma et al. [59] Male rats	rNSCs	35/155 days	1. No intervention (n = 13)	• Rotational behavior:
			2. Intranigral(SNc) 5×10^4^ rNSCs (n = 20)	1. Group without intervention: 233.9 ± 70.43
			3. Intrastriatal 5×10^4^ rNSCs (n = 5)	2. rNSCs SNc group:189.3 ± 63.24***
				3. rNSCs Intrastriatal group: 169.3 ± 47.28*
				• TH-positive cells in the SNc: 2 > 1
				• EGFP-labeled NSCs identified as TH^+^cells in 2 and 3
Ramos-Moreno, Castillo et al. [61] Female rats	hNSCs	45/165 days	1. Intrastriatal saline (n = 15)	• D-amphetamine-induced rotational behavior:
	hiNSCs		2. Intrastriatal 3×10^5^ hNSCs (n = 17)	1. Control group: 18 turns/min
	(expressing Bcl-X_L_)		3. Intrastriatal 3×10^5^ hiNSCs Bcl-X_L_ expression (n = 21)	2. hNSCs-treated group:17 turns/min
				3. hiNSCs-treated group: 3 turns/min***
				• Apomorphine-induced rotational behavior:
				1. Control group: 6.5 turns/min
				2. hNSCs-treated group: 2 turns/min**
				3. hiNSCs-treated group: 2.5/min**
				• Paw mobility test: 3** > 2* > 1

Abbreviations: *6-OHDA* 6 hydroxydopamine, *ASCs* Adult stem cells, *MSCs* Mesenchymal stem cells, *NSCs* Neural stem cells, *h* human *r* rat, *i* induced or transduced, *EGFP* Enhanced Green Fluorescent Protein, *BDNF* Brain-Derived Neurotrophic Factor, *GDNF* Glial cell Derived Neurotrophic Factor, *NT-3* Neurotrophine-3, *P* Pharmacologically active microcarriers, *Bcl-X*_*L*_ anti-apoptotic granulocyte-colony stimulating factor enhancing the expression of key genes involved in dopaminergic patterning, differentiation and maturation); *SNc* Substantia Nigra pars compacta, *TH*^*+*^ Tyrosine Hydroxylase Immunoreactive positive cells.

**Table 2:** Placebo-controlled stem cell applications in animal models of parkinsonism

***Striatal MPTP and subcutaneous proteasome inhibitor (MG-132) lesioned animals***				
**Authors animals**	**Product expanded allogenic ASCs Cyclosporin A**	**Applic/Observ time**	**Characteristics/Dosages/Application**	Outcome(* = p < 0.05 / ** = p < 0.01 / *** = p < 0.001)
Chao, He et al. [54]	mMSCs	Directly after last MPTP injection/1 month	1. Intraperitoneal saline (n = 24)	• SN TH^+^ cells: 3** > 2
			2. Intraperitoneal MPTP + IV saline (n = 24)	• SN microglial cells: 3* < 2
Male C57BL/6 mice			3. Intraperitoneal MPTP + IV 10^5^ mMSCs (n = 24)	Phagocytosis and Complement inhibition: 3* < 2
Park, Bang et al. [62]	hiMSCs	1 day after last MPTP and 3-NP injection/28 days	1. Intraperitoneal saline-treated (n = 10)	• Group 2 and 3: 48% loss of nigral cells;
			2. Intraperitoneal MPTP + 3-NP (n = 8)	• Group 3 compared to group 2:
Male C57BL/6 mice			3. Intraperitoneal MPTP + 3-NP and IV 1×10^6^ hiMSCs in 200 μl (n = 8)	a. 2% of hiMSCs in SN, and 4% in the Striatum
				b. Motor behavior improved* during 10 days
				c. Increased modulation of cell survival* and decreased modulation of death-signaling** pathways, with 20% cell survival**
				d. Decreased modulation of inflammation** and gliosis***, with a marked decrease of activated microglia** and astrocytes***
Bjugstad, Teng et al. [60]	hiNSCs	4 and/or 6 months after last MPTP injection/4 (n = 3) and 7 months (n = 4)	Bilateral intrastriatal and unilateral intranigral implantation of each 10^6^ hiNSCs (n = 7) in the intramuscular MPTP lesioned monkey	• >80% of hiNSCs immigrated along the impaired nigrostriatal pathway
African green Monkeys				• < 1% of a total of 2x10^6^ hiNSCs implanted within the caudate nucleus (intrastriatal) was identified at this site.
Park, Lee et al. [52]	hMSCs	21 days/3, 4, 6, 7, 10 weeks	1. MG-132 lesioned rats	• 1.7% hMSCs detected in the nigral substance
			2. MG-132 lesioned rats treated during 3 weeks with weekly intravasal application of 10^6^ hMSCs	• Survival of nigral and striatal TH^+^cells* after hMSCs
				• Increased striatal dopamine level* after hMSCs
Male rats				• Reduction* in microglia activation after hMSCs

Abbreviations: *ASCs* Adult stem cells, *IV* Intravenous, *MPTP* 1-methyl-4-phenyl-1,2,3,6-tetrahydropyridine, *MSCs* Mesenchymal stem cells, *NSCs* Neural stem cells, *h* human *m* mouse, *i* induced or transduced, *MG-132* carbobenzoxy-Leu-Leu-leucinal, a proteasome inhibitor. *SN* Substantia Nigra, *TH*^*+*^ Tyrosine Hydroxylase Immunoreactive positive cells.

As might be appreciated, in the studies reviewed in Tables 1 and 2, the following stem cells were used:

Expanded bone marrow-derived mesenchymal cells (of the mesodermal lineage) of rat [32,37,51-53], mouse [54] or human origin [55-58];

Expanded and enriched, or epigenetically induced bone marrow-derived mesenchymal stem cells [37,51,55,56], enabling functional development before actual transplantation;

Expanded neural stem cells of the ectodermal lineage, derived from the subventricular zone of the rat [59] or from a cultivated fetal human cell line [60,61].

But for three studies with intravenous [32,54] and/or intranasal [53] application, all stem cells are applied intranigral and/or intrastriatal.

Outcome measures comprised motor behavior (rotational behavior) as well as histological/histochemical measures. The main findings on the rotational motor behavior showed a significant reduction of turns/minute. But for 4/7 studies [32,51,53,58], in all studies with expanded MSCs [32,51,53,58], as well in all studies with expanded and enriched or epigenetically induced MSCs [37,51,55,56], and expanded neural stem cells [59,61], this reduction was established.

The administration of expanded, not-induced stem cells in these studies varied widely in relation to the onset of the toxin-induced motor parkinsonism. Stem cells were applied within 2 hours [32,55], within 5 to 14 days [57,58], or 30 days after the lesion [54,56,59-61]. Overlooking the effects in these studies on rotational behavior, the period in between application and lesioning seems to influence the clinical outcome: the earlier the application, the better the resulting clinical effects. Remarkably, after intravasal administration of ASCs, whether applied in the acute phase [32] or 3 weeks after lesioning [32], only a few ASCs (about 2%) could be detected in the nigral substance, the majority was trapped in the lungs. Nevertheless, also in these experiments, a significant beneficial effect on motor behavior could be established [32]. Here, it seems relevant to mention that after intrastriatal (intracaudate) application in the non-human primate MPTP model, 1% of the implanted stem cells could be identified at the injection site, whereas over 80% was found to be migrated to and along the impaired nigrostriatal pathways [60].

As for the histological/histochemical findings, after intracerebral stem cell application, in all experiments, more striatal TH-positive neurons were seen in the treated as compared to the non-treated, control lesioned animals, suggesting ASC-induced increased neuronal plasticity (neurorescue) with increased modulation of cell survival (and an increased striatal dopamine level), enhanced neurogenesis (progenitor cells in the subventricular zone), and a decreased modulation of inflammation, gliosis and death-signaling [51,52,55,57,59,62]. The same preservation of TH-positive cells was also observed after intravenous [32] and intranasal MSC application [53].

### Clinical experience with ASC in motor parkinsonism

As of yet, only three studies were reported, dealing with the effects of, intracerebral or intravasal applied, allogenic or autologous adult stem cells in patients suffering motor parkinsonism in PD, multiple system atrophy (MSA) or progressive supranuclear palsy (PSP) [63-65]. The results are summarized in Table 3. In most but not all PD patients, the subventricular application of both, allogenic and autologous bone marrow-derived mesenchymal stem cells did improve clinical scores of motor behavior, as expressed in a significant decrease of UPDRS scores in ON and OFF conditions. Interestingly, these effects did appear faster and more outspoken the earlier these interventions were applied, mimicking the results in preclinical experiments. Also Magnetic Resonance-tractography of degenerated dopaminergic projections did show significant improvements in those patients.

**Table 3:** Open label and placebo-controlled stem cell applications in clinical parkinsonism

**Authors Patients**	**Product**	**Observ. time (months)**	**Characteristics/** **Dosages/Application**	Outcome (* = p < 0.05 / ** = p < 0.01)
Venkataramana, Pal et al. [65]	Allogenic	3, 6, 12	1. PD patients with bilateral subventricular intracerebral application of 2×10^6^ /kg bodyweight MSCs in 2 ml (n = 8)	UPDRS in ON/OFF:
PD patients	MSCs		2. MSA + PSP patients with bilateral subventricular intracerebral application of 2×10^6^ /kg bodyweight MSCs in 2 ml (n = 4)	a. In PD patients: permanently improved* compared with baseline during both ON (18%: 51.2 versus 62.3) and OFF (31.2%: 59.5 versus 86.5). Effect stronger in patients with disease duration < 5 years (ON 45.5%/ OFF 56.7%) as compared to patients with a duration > 10 years (ON 6.3% OFF 12.4%).
MSA patients				b. Some MSA/PSP patients temporarily improved. The effect was not correlated with disease severity and disease duration. MR-tractography:
PSP patients				a. PD patients, after implantation, did show a trend of steadily improvement in tractographical images in genu and peduncles.
Open Label				b. MSA/PSP patients showed further reduction of tractographical images after stem cell implantation.
Venkataramana, Kumar et al. [64]	Autologous MSCs	10-36	PD patients (n = 7) with an UPDRS ON/OFF score 50.6/65.0 and a mean disease duration of 14.7 yr treated with 10^6^ MSCs/kg bodyweight in the subventricular zone	UPDRS in ON/OFF:
PD patients				a. In 3/7 patients there was a stable improvement of ON/OFF scores of 38% respect 22.9% with unchanged anti-parkinsonian medication.
Open Label				b. In 3/7 patients after treatment, only marginal clinical effects were observed
				Anti-parkinsonian medication significantly reduced in 2 patients.
Lee, kim et al. [63]	Autologous MSCs	1, 2, 3, 4, 5, 6, 8, 10, 12	Patients with Cognitive intact MSA-C (with UMSARS scores between 30 to 50)	UMSARS score
				a. MSCs-treated patients showed a reduced* increase of UMSARS score compared to placebo treated patients.
MSA patients			1. Placebo group: Intravenous or intra-arterial placebo (n = 17)	b. Intra-arterial application of MSCs was complicated with some MRI-detectable ischemic lesions
				Cognitive functions:
				Significantly* worsened in the placebo, but not in the MSCs-treated patients
Placebo controlled			2. MSCs group: 4×10^6^ MSCs intravenously or intra-arterial (n = 14)	MRI and FDG PET:
				Showed significantly increased* gray cerebral cortical areas respectively more decreased cortical and cerebellar glucose metabolism in placebo-treated, as compared to MSCs-treated patients.

Abbreviations: *MSCs* Mesenchymal stem cells, *MSA* multiple system atrophy, *MSA-C* multiple system atrophy, cerebellar type, *PSP* progressive supranuclear palsy, *MRI* magnetic resonance imaging, *UMSARS* unified MSA rating scale, *FDG PET* fluorodeoxyglucose positron emission tomography.

In MSA and PSP patients, compared to placebo-treated patients, in some patients, a temporary improvement [60] or reduced deterioration in motor and cognitive functions [60] witnessed a reduction in natural progressive deterioration with subventricular or intravasal ASC implantations. This reduced disease progression in these patients was also found reflected in MRI (Magnetic Resonance Imaging)- and FDG PET (fluorodeoxyglucose positron emission tomography)-imaging, showing less atrophy and less decreased glucose metabolism in cortex and cerebellum.

## Discussion

PD is a chronic progressive, diffuse α-synucleinopathic disease of the central nervous system in which (symptomatic) therapeutic strategies aim to compensate for the striatal dopamine deficiency in order to mainly decrease motor symptomatology. As of yet, protective/repairing therapeutic strategies in PD are an unmet need.

Generally speaking, stem cells, and specifically adult stem cells, pending their environment after re-implantation or pending their in-vitro induction, are supposed to not only differentiate into functional (neuronal) cells, including dopamine producing neurons, but to also easily expand and thus to deliver enough cells for transplantation. Although differentiation and proliferation of bone marrow-derived stem cells are jeopardized by aging and chronic diseases including diabetes [66], renal failure [67] and ALS (Amyotrophic lateral sclerosis) [68], in PD patients, up to the 15^th^ passage, ASCs are fully comparable to those of healthy age-matched individuals regarding phenotype, morphology and capacity to multi-differentiate [69], and are also able to inhibit T lymphocyte proliferation induced by mitogens.

Next to the multi-differentiated proliferative capacity, adult stem cells, and especially expanded and induced ASC’s, may secrete an array of trophic factors. As a matter of fact they may specialize to express mainly such factors by induction in vitro through exposure to trophic factors (including epidermic growth factor EGF and basic fibroblast growth factor bFGF), by transduction with a viral vector and/or by binding these ASCs to pharmacologically active microcarriers, containing trophic factors such as NT-3 prior to the application. When adding epidermal growth factor (EGF) and basic fibroblast growth factor (bFGF) to serum-free medium, in order to expand and induce human ASCs, the secretion of neurotrophic factors such as BDNF (normally neglectable) will increase to 125 ± 12 pg/day/10^6^ ASCs [70]. Thus induced ASCs will function as a vehicle for adequate delivery of neurotrophic factors (BDNF and GDNF) when transplanted in the central nervous system [55,70].

It is suggested that ASC-produced neurotrophic factors such as GDNF and BDNF enhance neuroprotection with an increased modulation of cell survival and a decreased modulation of inflammation, gliosis and death signaling. These trophic factors thus stimulate neurorescue by better protection of α-synucleinopathic jeopardized neurons, including DA producing neurons. This effect was also reported in a MPTP animal model in which allogenic neural stem cells were applied unilateral [71]. The jeopardized DA producing neurons were rescued and this effect was also observed in the opposite side of the SN in which no stem cells were applied [71]. So, in PD patients, autologous ASCs, and especially expanded and/or induced ASCs, due to their capacity to increase neuroplasticity, theoretically not only offer an increased neuroprotection, but also an increased neuro-repair in PD patients resulting in a slowing down of natural progression in this debilitating disease.

In preclinical studies, the stimulated secretion of GDNF and BDNF by ASCs is found to result in a higher number of striatal TH-positive neurons in the rat lesioned striatum as compared to placebo-treated rats, accompanied with a higher striatal dopamine level and decreased rotational behavior [58,70]. Other ASC-produced trophic factors, such as basic fibroblast growth factor bFGF and epidermal growth factor EGF, seem to be more relevant for neurorestoration (synthesis of extracellular matrix). In rodents, the intranigral application of (adenovirus mediated) glial cell line derived neurotrophic factor (GDNF), one week before the ipsilateral nigral 6-OHDA lesioning, did rescue 70% of the nigral TH-positive cells as compared to 30% in the not-pretreated, lesioned (control) animals, resulting in a significantly reduced rotational behavior: 5.4 ± 15.2 turns/15-minutes versus 40.8 ± 25 turns/15-minutes (p < 0.05) [72]. Note however that each intracerebral needle-induced manipulation per se might initiate the secretion of the same neurotrophic factors and/or cytokines mimicking a clinical effect, which may confound the results of a placebo treatment [73,74].

In the preclinical studies, reviewed above, two studies were performed in which ASCs were locally applied within 2 hours after the application of a 6-OHDA unilateral lesion in rodents [32,55]. Both studies, reported a significant improvement in rotational behavior and in survival of TH positive neurons within 4–6 weeks. In the studies applying ASCs, and especially expanded non-induced ASCs, later on, in a more chronic phase of a lesion, no significant reduction in rotational behavior could be established [28]. In translation, the clinical effects of implantation of expanded MSCs in PD patients might profit their application in an early phase of their disease. Indeed, in PD patients, the clinical effects of stem cell application seem to relate with disease duration [60].

Finally, as ASCs are large cells, unable to cross the blood–brain barrier [44], intracerebral application seems to be superior to intravasal application, as only 1-2% of thus applied cells will reach the central nervous system [52]. Intranasal administration of MSCs, however, might offer an alternative, as this way of application in unilateral 6-OHDA-lesioned rodents did result in both, a significantly improved motor behavior and a significant decrease in inflammatory cytokines (suggesting a strong cell protective effect by inhibiting inflammation cascades) with 24% of the cells surviving for at least 4.5 month in the central nervous system [53].

## Conclusions

In conclusion

ASCs are capable of migrating to the lesioned cells/organs throughout the body, a phenomenon, which is called homing.

ASCs are easily to harvest out of the iliac crest. Their number might be increased by expanding them, and their functions might be developed prior to re-application by inducing them. Unlike ASCs in patients with other chronic diseases, in PD patients those cells are not impaired.

Expanded and/or induced ASCs may stabilize motor (and non-motor) parkinsonism in patients suffering PD and may be also Parkinson-plus syndromes, by increasing neural plasticity rather than by differentiating into neurons. Early in the disease, ASCs will promote neurorescue/neuroprotection by increasing immune modulation and reducing inflammation), especially when expanded (undifferentiated) ASCs are administered. Later in the disease, they mainly will promote neurorestoration, especially when induced (differentiated) ASCs are given.

Neurotrophic factors such as BDNF and GDNF (mainly interfering with destructive pathways) play a major role in neuroprotection, whereas other growth factors such as EGF and bFGF (promoting synthesis of extracellular matrix) are more important in neurorepair. The best time to start with ASCs administration is in the very early phase of Parkinson’s disease.

## Competing interests

The authors declare that they have no competing interests.

## Authors’ contributions

JdM compiled the article and did the literature search and summarized the preclinical and clinical studies as a part of his PhD. JDM wrote the draft and invited the 2 co-authors to comment and revised the draft. EW is the promoter of JdM and revised the article critically and was also responsible for the completeness of the issues addressed in this article. CL as an experienced neurologist familiar with Parkinsonism revised the article to assure the translational aspects of the content and was especially responsible for revising the clinical part of the article. All authors read and approved the final manuscript.
